# Evaluation of Apoptosis-Inducing Coumarins Isolated from *Peucedanum japonicum* Roots: The Potential for Leukemia Treatment via the Mitochondria-Mediated Pathway

**DOI:** 10.3390/cells13231982

**Published:** 2024-11-29

**Authors:** Kyung-Yun Kang, Sonny C. Ramos, Sung-Ju Lee, Sang-Jip Nam, Jong-Jin Kim

**Affiliations:** 1 R&D Team, Suncheon Research Center for Bio Health Care, Suncheon 57962, Republic of Korea; nms-kang@nate.com; 2 Department of Biomedical Science, Sunchon National University, Suncheon 57922, Republic of Korea; ynnosomarc@gmail.com; 3 KM Convergence Research Division, Korea Institute of Oriental Medicine, Yuseong-daero 1672, Yuseong-gu, Daejeon 34054, Republic of Korea; sungjulee@kiom.re.kr; 4 Department of Chemistry and Nanoscience, Ewha Womans University, Seoul 03760, Republic of Korea; 5 Glocal University Project Team, Sunchon National University, Suncheon 57922, Republic of Korea

**Keywords:** *Peucedanum japonicum*, apoptosis, mitochondrial membrane potential, caspase

## Abstract

Inducing programmed cell death in tumors is a fundamental approach in cancer therapy, prompting extensive efforts to discover bioactive compounds with anticancer properties. *Peucedanum japonicum*, a plant used in traditional medicine across East Asia, has been reported to exhibit various biological activities, including anticancer effects. This study aimed to evaluate the apoptosis-inducing effects of methanol/dichloromethane (MeOH/CH_2_Cl_2_) extracts of *P. japonicum* roots and their components in HL-60 human leukemia cells. Compounds were isolated using solvent extraction and reverse-phase column chromatography, and their structures were confirmed by nuclear magnetic resonance (NMR) spectroscopy. The cytotoxicity effect of the compounds was tested on various cancer cell lines (HL-60, A549, and MCF-7). Two coumarins, (−)-isosamidin (**1**) and 3′S,4′S-disenecioylkhellactone (**2**), were isolated through bioactivity-guided fractionation. Compound **2** significantly induced apoptosis in HL-60 cells, as evidenced by an increase in the sub-G1 cell population and the initiation of both early and late apoptosis. Additional apoptotic markers, including reduced mitochondrial membrane potential (MMP) and increased cleavage of caspase-3, -8, and -9, were observed. These findings suggest that compound **2** shows potential as a candidate for leukemia treatment, providing a promising natural-product-based approach to cancer therapy.

## 1. Introduction

Cancer occurs due to uncontrolled cellular proliferation, and despite advances in cancer therapy, many tumors ultimately metastasize. The incidence of cancer continues to rise worldwide, and although new drugs are continuously being developed, many cancer types still have high mortality rates. To overcome cancer, combination or target-specific treatments are emerging as promising strategies, often using chemotherapy, which involves chemical substances derived from natural or synthetic products [1,2]. However, one of the weaknesses of chemotherapy is its nonspecific cytotoxicity in the body. In the quest to develop new anticancer agents with fewer side effects, herbal medicines continue to be investigated, as they have been used for centuries. A prominent example is plant-derived natural products, such as flavonoids, terpenoids, and alkaloids, which have received considerable attention in recent years due to their diverse pharmacological properties, including cytotoxic and anticancer effects [3,4].

*Peucedanum japonicum* (Umbelliferae) is a coastal plant widely distributed in Japan, the Philippines, China, and Korea, and mainly grows in sandy soil or sandy loam. Its root is used in folk medicine for the treatment of coughs, colds, and headaches and as an anodyne [5]. The representative natural products previously isolated from *P. japonicum* include pyranocoumarins, phenylcoumarins, and other coumarin derivatives, which possess effects including the inhibition of the Ca^2+^ channel, monoamine oxidase, and nitric oxide synthase activities. In addition, *P. japonicum* extract has biological activity such as antidiabetic, anti-inflammatory, anticonvulsant, and blood-pressure-lowering effects, as well as ameliorative effects on bronchial asthma [6,7,8,9,10,11,12,13,14,15,16,17,18,19,20].

The induction of apoptotic cell death in tumors is a promising strategy for eliminating cancer, and the most important point is the development of cancer-specific active molecules that do not exhibit cytotoxicity in normal somatic cells. Apoptotic cell death is a complex process involving biochemical events and well-defined morphological changes, such as cell shrinkage, cytoplasmic vacuolization, chromatin condensation, DNA fragmentation, and dissipation of mitochondrial membrane potential (MMP) [2,21,22]. Recent studies have documented that two major pathways regulate apoptosis: the extrinsic pathway, related to cell surface receptors, and the intrinsic pathway, mediated by mitochondria [21]. The aforementioned studies have also demonstrated that mitochondria play a critical role in apoptosis progression [23,24,25]. Mitochondrial permeability transition leads to the loss of MMP, causing the translocation of the proapoptotic protein Bax to the mitochondria and the release of cytochrome C from the mitochondria into the cytosol, which subsequently results in the activation of apoptotic caspase cascades [24]. Compounds **1** and **2**, specifically isosamidin and 3′S,4′S-disenecioylkhellactone, have been previously isolated and studied [15,26,27,28,29,30]. These compounds are part of the diverse coumarin derivatives isolated from *P. japonicum*, which include pyranocoumarins, phenylcoumarins, and other related structures. Both isosamidin and 3′S,4′S-disenecioylkhellactone have shown potential in cancer research across different cell types; however, their effects in leukemia cells specifically have not been investigated in detail.

This study reports the anticancer effects of the pure compound 3′S,4′S-disenecioylkhellactone (compound **2**), isolated from *P. japonicum*, in inducing apoptosis in leukemia cells. Specifically, compound **2**, at a concentration of 30 μM, was shown to promote cell death in HL-60 cells, which are derived from peripheral blood leukocytes in the context of acute promyelocytic leukemia [31].

## 2. Materials and Methods

### 2.1. Plant Materials

*P. japonicum* roots (harvested from Geumo-do, Nam-myeon, Yeosu-si, Jeollanam-do, Republic of Korea) were purchased from the Dongbu Herbal Farming Cooperative (Suncheon, Republic of Korea) in January 2015. After verifying the certificate of origin, the experiment was conducted using the plant specimen ADNA200911292195 from the National Institute of Biological Sciences for comparison.

### 2.2. Chemicals and Reagents

RPMI 1640 was purchased from Thermo Scientific (Waltham, MA, USA); Dulbecco’s Modified Eagle medium (DMEM) was obtained from Gendepot (Baker, TX, USA); the Cell Counting Kit-8 (CCK-8) was procured from Dojindo Laboratories (Kumamto, Japan); dimethyl sulfoxide (DMSO) and propidium iodide (PI) were acquired from Sigma Aldrich (St. Louis, MO, USA); Annexin V-FITC was purchased from BD Biosciences (Franklin Lakes, NJ, USA); and the JC-1 Assay Kit was obtained from Invitrogen (Carlsbad, CA, USA). Methanol, dichloromethane, and hexane (Duksan Co., Ansan, Republic of Korea); HPLC-grade acetonitrile, water, and methanol (J. T Baker, Phillipsburg, NJ, USA); chloroform-d (Cambridge Isotope Laboratories, Inc., Tewksbury, MA, USA); silica gel 60 (70–230 mesh, Merck Co., Kenilworth, NJ, USA); Luna 5u (C18 100A 250 × 10 mm, Phenomenex Inc., Torrance, CA, USA); and YMC-actus (Triart C18 250 × 20 mm, YMC, Kyoto, Japan) were also used.

### 2.3. Extraction and Isolation of Compounds from P. japonicum

**Figure 1:**
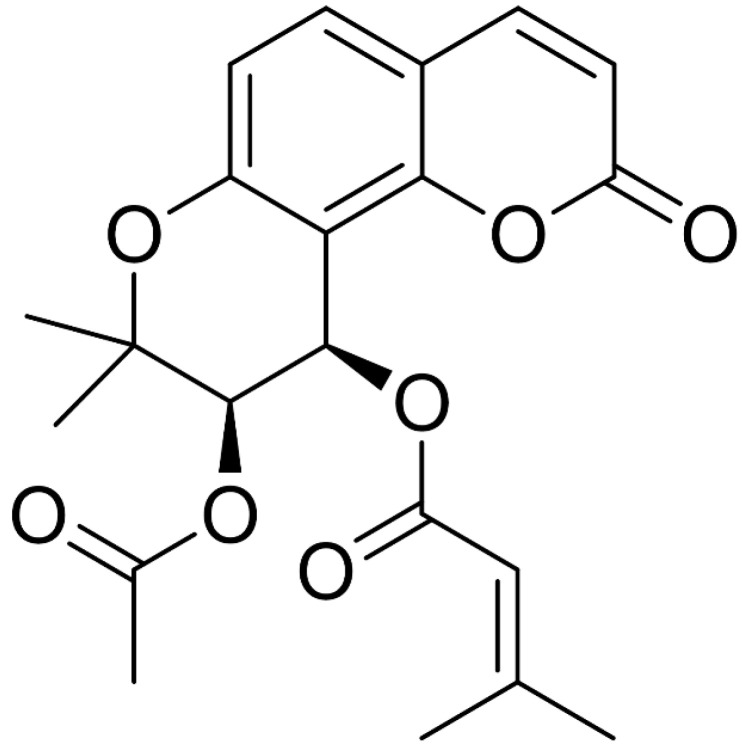
Structure of compound **1**.

**Figure 2:**
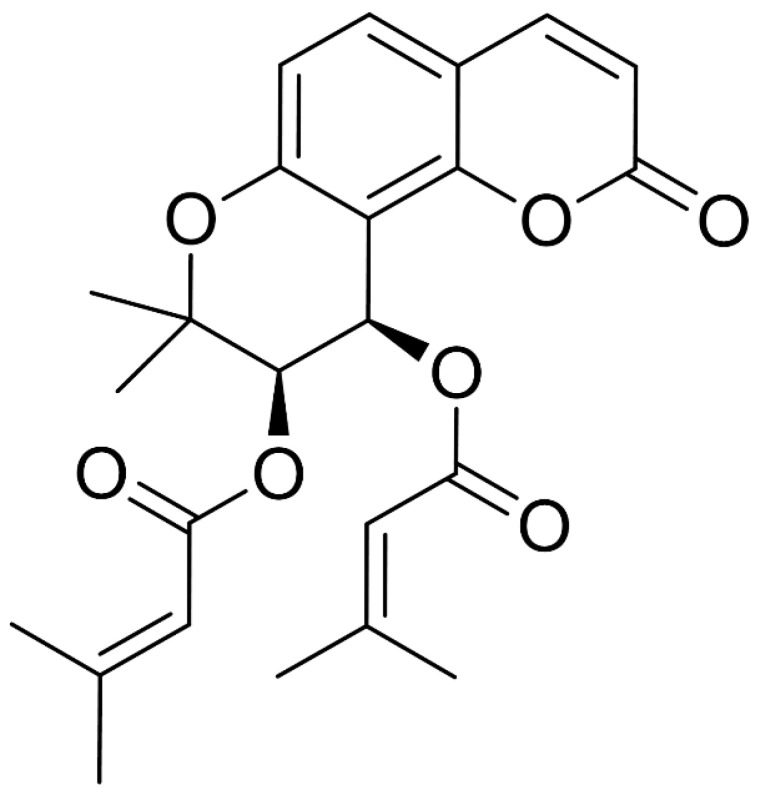
Structure of compound **2**.

The roots of *P. japonicum* (dry wt. 1.0 kg) were extracted three times with MeOH in CH_2_Cl_2_ (1:1, *v*/*v*) at room temperature. The separation process is illustrated in Figure S1. These extracts were combined and partitioned three times between MeOH and *n*-hexane. The MeOH layer was further partitioned between H_2_O and ethyl acetate to establish an H_2_O fraction and an ethyl acetate fraction (8.41 g). The ethyl acetate-soluble fraction was subjected to silica gel flash column chromatography and eluted with a step gradient solvent system of 100% to 0% CH_2_Cl_2_ in MeOH, resulting in 11 fractions (1–11) (Figure S2). Compounds **1** (1.0 mg, tR 20.7 min) and **2** (1.2 mg, tR 29.5 min) were obtained by reverse-phase HPLC (Shiseido CAPCELL C_18_ 5 μm, 250 × 10 mm, 2.0 mL/min, UV detection at 210 nm) using 70% CH_3_CN in H_2_O as the eluent. Compounds **1** and **2** were identified as (−)-isosamidin and 3′S,4′S-disenecioylkhellactone, respectively, by comparing their spectroscopic data and optical rotations with previously reported data [32,33]. For (−)-isosamidin (1), ^1^H NMR (CDCl_3_, 700 MHz) δ 7.61 (1H, d, *J* = 9.5), 7.36 (1H, d, *J* = 8.7), 6.81 (1H, d, *J* = 8.5), 6.57 (1H, d, *J* = 4.8), 6.23 (1H, d, *J* = 9.5), 5.69 (1H, s), 5.35 (1H, d, *J* = 4.8), 2.17 (3H, d, *J* = 1.2), 2.11 (3H, s), 1.90 (3H, d, *J* = 1.2), 1.45 (3H, s), 1.41 (3H, s); ^13^C NMR (CDCl_3_) δ169.5 (C), 164.9 (C), 159.5 (C), 158.4 (C), 156.4 (C), 153.6 (C), 128.7 (CH), 114.8 (CH), 114.1 (C), 112.8 (CH), 112.3 (C), 106.7 (C), 77.7 (C), 68.7 (CH), 27.4 (CH^3^), 24.5 (CH_3_), 23.1 (CH_3_), 20.6 (CH_3_), 20.3 (CH_3_); LRESIMS m/z 286.4 (M − 99 + H)^+^, 793.3 (2M + Na)^+^ (^13^C NMR chemical shifts were determined using 2D NMR spectroscopy data and confirmed by comparing them with previously reported data). The optical rotation for compound ([α]_D_^25^ = −67°) indicated the 3′*R*, 4′*R* configurations (Figure 1 and Figure S4). For 3′S,4′S-disenecioylkhellactone (2), ^1^H NMR (CDCl_3_, 700 MHz) δ7.60 (1H, d, *J* = 9.2), 7.37 (1H, d, *J* = 8.7), 6.82 (1H, d, *J* = 8.7), 6.65 (1H, d, *J* = 4.8), 6.24 (1H, d, *J* = 9.5), 5.69 (1H, s), 5.64 (1H, s), 5.37 (1H, d, J = 4.8) 2.22 (3H, s), 2.17 (3H, s), 1.91 (3H, s), 1.90 (3H, s), 1.48 (3H, s), and 1.44 (3H, s); ^13^C NMR (CDCl_3_) δ 167.5 (C), 167.3 (C), 162.7 (C), 160.8 (C), 160 (C), 158.8 (C), 155.9 (C), 146.6 (CH), 131.7 (CH), 117.5 (CH), 117.4 (CH), 114.8 (CH), 114.4 (CH), 113.6 (C), 109.4 (C), 79.4 (C), 72.0 (CH), 61.8 (CH), 28.3 (CH_3_), 28.2 (CH_3_), 26.5 (CH_3_), 23.2 (CH_3_), 21.3 (CH_3_), 21.2 (CH_3_); LRESIMS m/z 327.1 [M − 100 + H]^+^, 876.3 [2M + Na]^+^ (^13^C NMR chemical shifts were determined using 2D NMR spectroscopic data and confirmed by comparing them with previously reported data). The optical rotation for compound ([α]_D_^25^ = +12°) indicated the 3′*R*, 4′*R* configurations (Figure 2 and Figure S5).

### 2.4. Cell and Cell Culture

Human acute promyelocytic leukemia cells (HL-60; KCLB 10240), human lung adenocarcinoma cells (A549; KCLB 10185), and human breast adenocarcinoma cells (MCF-7; KCLB 30022) were obtained from the Korean Cell Line Bank (Seoul, Republic of Korea) and cultured in RPMI 1640 supplemented with 10% heat-inactivated fetal bovine serum (FBS), penicillin (100 U/mL), and streptomycin (100 μg/mL). Madin–Darby canine kidney (MDCK; KCLB 10034) cells were also obtained from the Korean Cell Line Bank and cultured in DMEM containing 10% FBS, 100 U/mL penicillin G, and 100 μg/mL streptomycin sulfate. The purities of all compounds tested by HPLC were confirmed to be >95%. Samples of test compounds were dissolved in DMSO and added to the medium for a final concentration of 0.03%. Cultures were maintained at 37 °C under 5% CO_2_/95% air, and media were changed every two days.

### 2.5. Cytotoxicity Assay

Cell viabilities were determined using the Cell Counting Kit-8 (CCK-8) assay. First, MCF-7, A549, AGS, and HL-60 cells were suspended in RPMI 1640 medium at densities of 3 × 10^4^ cells/mL, 1 × 10^4^ cells/mL, 2 × 10^4^ cells/mL, and 1 × 10^5^ cells/mL, respectively. Next, 100 μL of the cell suspensions were added per well to 96-well flat-bottomed microtiter plates, followed by 100 μL of compounds **1** and **2,** to achieve final concentrations of 1, 3, 10, and 30 μM. The plates were then incubated for 24 h at 37 °C in 5% CO_2_/95% air. Second, HL-60 cells were resuspended in RPMI 1640 medium at 1 × 10^5^ cells/mL, whereas MDCK cells were suspended in DMEM at 1 × 10^4^ cells/mL. Next, 100 μL of cell suspension was added to the wells of 96-well flat-bottomed microtiter plates, followed by 100 μL of compounds **1** and **2** (to final concentrations of 1, 10, and 30 μM), and the plates were incubated for 24 h at 37 °C in 5% CO_2_/95% air. Three replicates per condition were used in the experiments. After 24 h, 100 μL of the medium was replaced with an equal volume of fresh medium containing 10 μL of CCK-8, after which cell viability was measured.

### 2.6. Cell Cycle Assay

To determine the cell cycle distribution, HL-60 cells (5 × 10^5^/well) were seeded and treated with compound **1** or **2** at a concentration of 30 μM for 12, 18, and 24 h. After incubation, the cells were harvested, fixed in 70% ethanol, and treated with ribonuclease A to increase cell permeability. The cell pellets were then incubated with PI for 2 h at 4 °C in the dark before being analyzed by flow cytometry. The percentages of cells in the G0/G1, S, and G2/M phases of the cell cycle, as well as the sub-G1 peak, were determined after excluding cell debris and aggregates.

### 2.7. Annexin V/PI Assay of Apoptotic Cells

Apoptosis induced by compounds **1** and **2** was quantified by flow cytometry using Annexin V-FITC and propidium iodide (PI) solution according to the manufacturer’s instructions. Briefly, HL-60 cells (5 × 10^5^/well) were seeded into 24-well plates and treated with compound **1** or **2** (both at 30 µM) for 12, 18, and 24 h. Apoptosis was analyzed by staining with Annexin V-FITC/PI, followed by flow cytometry. The percentages of apoptosis were calculated by counting the number of Annexin V- and PI-positive cells.

### 2.8. Detection of Changes in Mitochondrial Membrane Potentials (MMPs)

HL-60 cells were seeded in 24-well plates at a density of 5 × 10^5^ cells/mL and treated with compounds **1** or **2** at a concentration of 30 μM for various periods (12, 18, and 24 h). The cells were then harvested and stained with the lipophilic dye JC-1 (5,5′,6,6′-tetrachloro-1,1′,3,3′-tetraethylbenzimidazole-carbocyanine iodide), which was used to measure the MMP by flow cytometry. When JC-1 enters the mitochondria, it aggregates and fluoresces red. Upon loss of MMP, the dye diffuses throughout the cytoplasm and fluoresces green. The histogram displays the population of cells showing green (JC-1 monomers) and red (JC-1 aggregates) fluorescence. Frequency plots were prepared with FITC and PE gating to determine the percentages of mitochondria stained green (indicating a loss of MMP) and red (indicating normal MMP) [34].

### 2.9. Activation of Caspase-3, -8, and -9

The activities of caspase-3, -8, and -9 were determined using the caspase-3, -8, -9 activity kit according to the manufacturer’s protocol. HL-60 cells were seeded into 24-well plates and treated with compounds **1** and **2** at a concentration of 25 μM for 12, 18, and 24 h. The cells were then collected, washed, fixed, and stained with FITC before being analyzed by flow cytometry.

### 2.10. Statistical Analysis

Differences between groups are presented as the means ± S.D. of three replicates. Statistical differences were analyzed using Student’s *t*-test. Probability values less than 0.05 was considered significant (*p*-values * < 0.05, ** < 0.01, *** < 0.001 vs. control, ^#^ < 0.05, ^##^ < 0.01, ^###^ < 0.001 vs. 24 h sample).

## 3. Results

### 3.1. Isolation of Compounds from the Roots of P. japonicum

Dried and pulverized roots of *P. japonicum* were submerged in a MeOH/CH_2_Cl_2_ (1:1, *v*/*v*) mixture at room temperature to obtain an extract, which was then fractioned into *n*-hexane, ethyl acetate, and H_2_O fractions as described in the Materials and Methods. The ethyl acetate fraction was further subjected to repeated column chromatography followed by RP-HPLC, yielding two coumarins: (-)-isosamidin (**1**) and 3′S,4′S-disenecioylkhellactone (**2**). The structures of these compounds were unequivocally determined by 1D and 2D NMR and by comparing the spectroscopic data with literature values (Figure 1, Figure 2 and Figures S3–S5). Table S1 correlates the chemical shift values (δH and δC) of compounds **1** and **2** with their respective hydrogen and carbon atoms (Figures S6–S12).

Compound **1** was obtained as a colorless gum, showing ionic peaks at m/z 286.4 [M − 99 + H]^+^ and 793.3 [2M + Na]^+^ in the LRMS. The NMR spectrum of compound **1** displayed proton signals at δH 7.61 (1H, d, *J* = 9.5), 7.36 (1H, d, *J* = 8.7), 6.81 (1H, d, *J* = 8.5), 6.57 (1H, d, *J* = 4.8), 6.23 (1H, d, *J* = 9.5), 5.69 (1H, s), 5.35 (1H, d, *J* = 4.8), 2.17 (3H, d, *J* = 1.2), 2.11 (3H, s), 1.90 (3H, d, *J* = 1.2), 1.45 (3H, s), 1.41 (3H, s) and carbon signals at δC 169.5 (C), 164.9 (C), 159.5 (C), 158.4 (C), 156.4 (C), 153.6 (C), 128.7 (CH), 114.8 (CH), 114.1 (C), 112.8 (CH), 112.3 (C), 106.7 (C), 77.7 (C), 68.7 (CH), 27.4 (CH_3_), 24.5 (CH_3_), 23.1 (CH_3_), 20.6 (CH_3_), and 20.3 (CH_3_). Based on the interpretation of MS and 1D NMR spectroscopic data, as well as a comparison of the NMR data with previous reports [32,33], compound **1** was identified as (−)-isosamidin. Meanwhile, Chen et al. have reported (−)-isosamidin with the following findings: colorless needles, m.p. 120 122 °C (*n*-hexane), [α]25D: –71.6° (*c* 0.08, CHCl_3_), EI MS *m*/*z* (%): 386 ([M]^+^, 0.3), 355 (7), 326 (4), 311 (10), 244 (7), 229 (38), 83 (100). IR *ν* KBr max cm^−1^: 1741 (C=O). UV λ MeOH max nm (log ε): 222 (4.93), 255 (4.36), 300 sh (4.63), 323 (4.81). ^1^H-NMR (CDCl_3_, 400 MHz): δ 1.42 (3H, *s*, Me-2′), 1.46 (3H, *s*, Me-2′), 1.89 (3H, *d*, *J* = 1.2 Hz, H 4‴), 2.09 (3H, *s*, H-2″), 2.23 (3H, *d*, *J* = 1.2 Hz, H-5‴), 5.30 (1H, *d*, *J* = 4.8 Hz, H-3′), 5.64 (1H, *q*, *J* = 1.2 Hz, H-2‴), 6.22 (1H, *d*, *J* = 9.6 Hz, H-3), 6.58 (1H, *d*, *J* = 4.8 Hz, H-4′), 6.79 (1H, *d*, *J* = 8.8 Hz, H-6), 7.34 (1H, *d*, *J* = 8.8 Hz, H-5), and 7.58 (1H, *d*, *J* = 9.6 Hz, H-4) [35].

Compound **2** is a derivative of compound **1**, sharing the same structural skeleton except for the substitution of a senecioyl group at the 3′-carbon. Based on a comparison of the NMR data with previously reported literature [32,33], compound **2** was identified as 3′S,4′S-disecenecioylkhellactone.

### 3.2. Cytotoxic Effect of Isolated Compounds ***1*** and ***2*** on the Cancer Cell Lines

This study represents a preliminary investigation into the effects of coumarins extracted from *P. japonicum* on cancer cells, specifically targeting leukemia (HL-60) cells. Currently, no studies have explored the apoptotic effects of coumarins from this plant on leukemia cells. However, previous research by Suzuki et al., Chun et al., Do et al., and Park et al. has demonstrated the pharmacological effects of these compounds against human gastric cancer cells and other diseases using concentrations ranging from 1 to 100 µM [27,28,29,30]. We used these studies as a basis for selecting compound concentrations for leukemia cell treatment.

**Figure 3:**
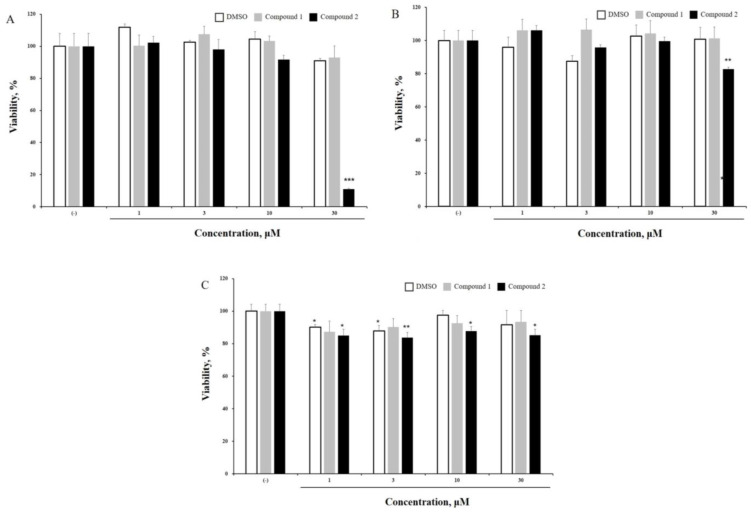
The effect of *P. japonicum* root extracts on the viability of cancer cell lines. Three cancer cell lines ((**A**): human acute promyelocytic leukemia cells, HL-60; (**B**): human lung adenocarcinoma cells, A549; (**C**): human breast adenocarcinoma cells, MCF-7) were treated with *P. japonicum* extracts for 24 h. Culture supernatants were removed, and Cell Counting Kit-8 (CCK-8) was added to the cells. Viability was quantified using an ELISA reader. The results are presented as the means ± S.D. of experiments performed in triplicate. * *p* ≤ 0.05, ** *p* ≤ 0.01, *** *p* ≤ 0.001.

**Figure 4:**
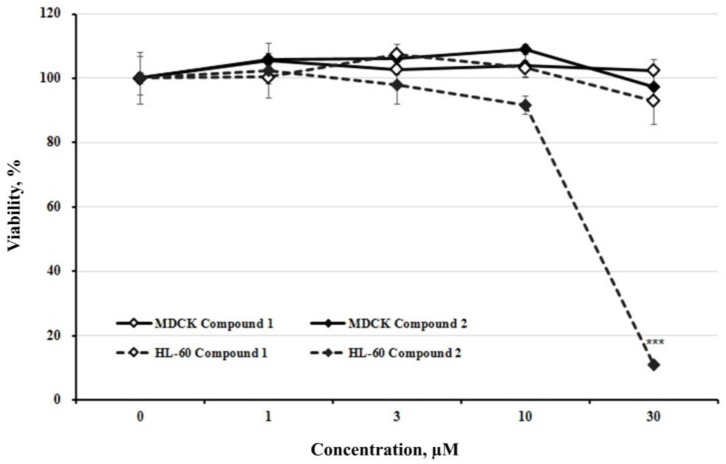
HL-60 cell viability versus MDCK cell viability. HL-60 and MDCK cells were treated with each dose of compounds **1** and **2** for 24 h. The culture supernatant was removed, and CCK-8 was added. Cell viabilities were determined by ELISA. The results are presented as the means ± S.D. of experiments performed in triplicate. *** *p* ≤ 0.001.

To evaluate cytotoxicity, three different cancer cell lines (MCF-7, A549, and HL-60) were treated with compounds **1** and **2** for 24 h. The viability of MCF-7 and A549 cells did not change significantly after treatment with compounds **1** and **2** (Figure 3B,C). However, compound **2** (30 µM) reduced the viability of leukemia cell line HL-60, to 11.077 ± 0.520% (Figure 3A). Various anticancer studies have focused on reducing cytotoxic effects on normal cells. Therefore, we examined the effects of compounds **1** and **2** on MDCK cells (normal kidney cells). HL-60 and MDCK cells were treated with compounds **1** or **2** (1, 10, and 30 μM) for 24 h, and cell viability was assessed. As shown in Figure 4, cytotoxicity was observed only in HL-60 cells treated with compound **2** (30 μM). Interestingly, compound 2 did not exert any cytotoxic effects on MDCK cells (treated, 97.229 ± 3.818%). Meanwhile, compound **1** did not show significant cytotoxicity in either HL-60 or MDCK cells (HL-60-treated, 92.909 ± 7.244%; MDCK-treated, 102.480 ± 3.319%) (Figure 3). Moreover, the IC_50_ values for the HL-60 cell line are 634.8 μM for compound **1** and 20.21 μM for compound **2**; for the A549 cell line, they are N.D. (not determined) for compound **1** and 168.2 μM for compound **2**; and for the MCF-7 cell line, they are 277.6 μM for compound **1** and 122.3 μM for compound **2**. GraphPad Prism 5.0 was used to calculate the IC_50_ values of the tested compounds.

### 3.3. Increase in the Apoptotic Population by Compound ***2*** in Cell Cycle Analysis

**Figure 5:**
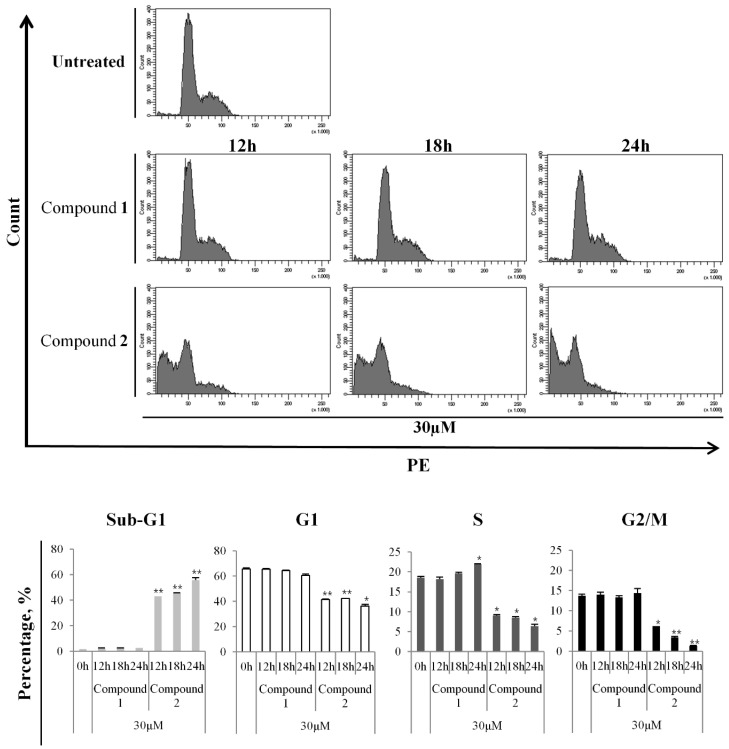
Incremental increases in the percentage of HL-60 cells in the sub-G1 phase after treatment with compound **2**. HL-60 cells were treated with compound **2** for 12, 18, and 24 h. After the indicated incubation times, cells were harvested, stained with PI for 2 h, and analyzed by flow cytometry. The results are presented as the means ± S.D. of experiments performed in duplicate. * *p* ≤ 0.05, ** *p* ≤ 0.01.

To determine whether the cytotoxic effect of compound **2** on HL-60 cells was associated with apoptosis, we investigated time-dependent changes in the proportions of cells in the sub-G1 phase by flow cytometry (Figure 5). Compound **2** induced apoptosis in HL-60 cells, and 24 h of treatment shifted more cells to the sub-G1 phase (untreated, 1.9 ± 0.0%; compound **2** (30 µM), 55.7 ± 1.84%). Consistent with the above data, the increase in the sub-G1 population following treatment with compound **2** reduced percentages of cells in the G1 (untreated, 65.55 ± 0.92%; compound **2** (30 µM): 12 h, 41.45 ± 0.35%; 18 h, 42.15 ± 0.07%; 24 h, 36.25 ± 1.34%), S (untreated, 18.5 ± 0.42%; compound **2** (30 µM): 12 h, 9.05 ± 0.21%; 18 h, 8.55 ± 0.21%; 24 h, 6.35 ± 0.49%), and G2/M (untreated, 13.75 ± 0.35%; compound **2** (30 µM): 12 h, 6.2 ± 0.00%; 18 h, 3.45 ± 0.21%; 24 h, 1.3 ± 0.14%) phases (Figure 5). These results suggest that compound **2** strongly induced cell cycle arrest at the sub-G1 phase in HL-60 cells.

### 3.4. Induction of Apoptotic Cell Death by Compound ***2***

**Figure 6:**
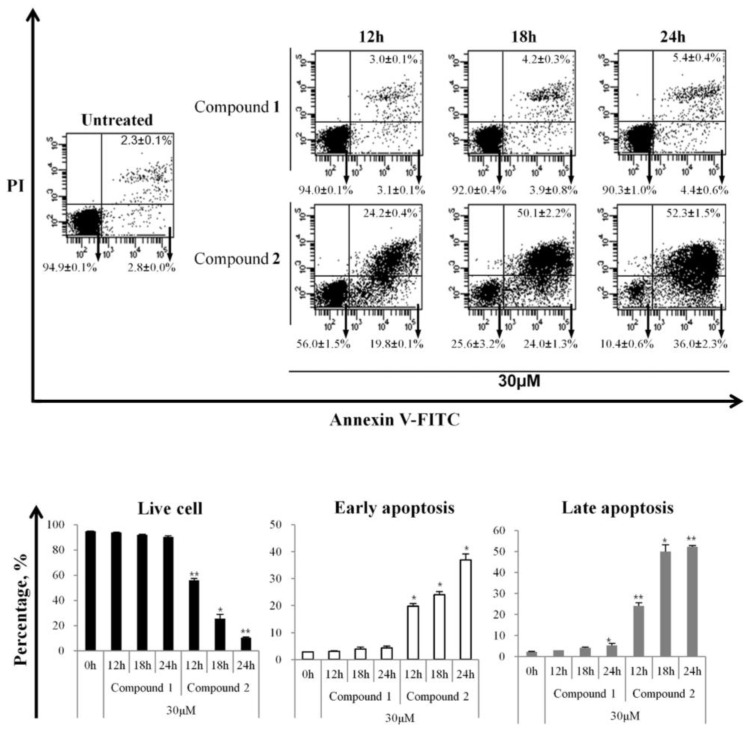
The effect of compound **2** on HL-60 cell membranes. HL-60 cells were treated with compound **2** for 12, 18, and 24 h. After the indicated times, cells were harvested, stained with Annexin V (FITC) and PI for 15 min, and analyzed on a flow cytometer. The results are presented as the means ± S.D. of experiments performed in duplicate. * *p* ≤ 0.05, ** *p* ≤ 0.01.

The induction of early and late apoptosis through membrane collapse was evaluated (Figure 6). Apoptosis was quantified using Annexin V/PI staining, which detects phosphatidylserines on the external layer of the cell membrane, a common feature of apoptosis. As shown in Figure 6, the percentages of early and late apoptotic cells increased in a time-dependent manner in response to compound **2** (30 μM). In contrast, compound **2** induced a time-dependent decrease in the live cell populations. These data indicate that HL-60 cell death induced by compound **2** is clearly linked to the apoptotic pathway.

### 3.5. Changes of MMP by Treating Compound ***2***

**Figure 7:**
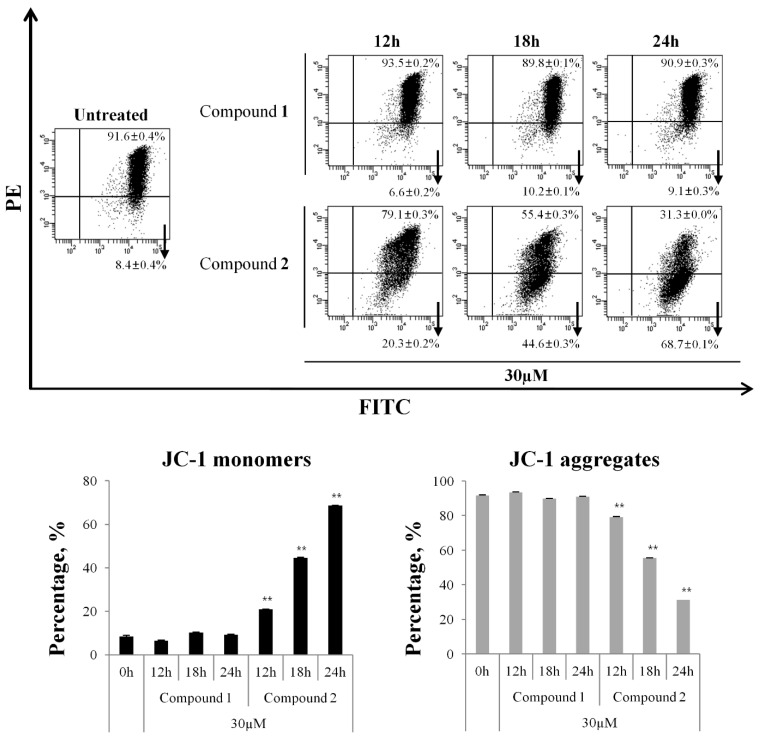
The effect of compound **2** on mitochondrial membrane potential in HL-60 cells. HL-60 cells were treated with compound **2** for 12, 18, and 24 h. After the indicated incubation times, cells were harvested, stained with JC-1 for 30 min, and then analyzed by flow cytometry. The results are presented as the means ± S.D. of experiments performed in duplicate. ** *p* ≤ 0.01.

Initiating and progressing apoptosis in a cell require intracellular events before morphology changes occur in early and late apoptosis. To confirm the early stage of apoptosis induced by compound **2** at the intracellular level, we evaluated the MMP using the JC-1 kit and analyzed the samples with a flow cytometer. Live cells with functional mitochondria showed red JC-1 aggregates, whereas apoptotic cells with impaired mitochondria contained green JC-1 monomers. Green and red fluorescence intensities were measured in HL-60 cells treated with compounds **1** or **2** for different amounts of time (12, 18, and 24 h). Compared with the untreated control, compound **2** increased JC-1 green fluorescence and decreased JC-1 red fluorescence in a time-dependent manner (Figure 7). On the other hand, compound **1** did not induce changes in all incubation hours. Thus, compound **2** increased the permeability of the mitochondrial membrane in HL-60 cells, initiating apoptosis via the mitochondria-mediated pathway.

### 3.6. Activation of the Caspase Family by Compound ***2*** in Apoptosis

**Figure 8:**
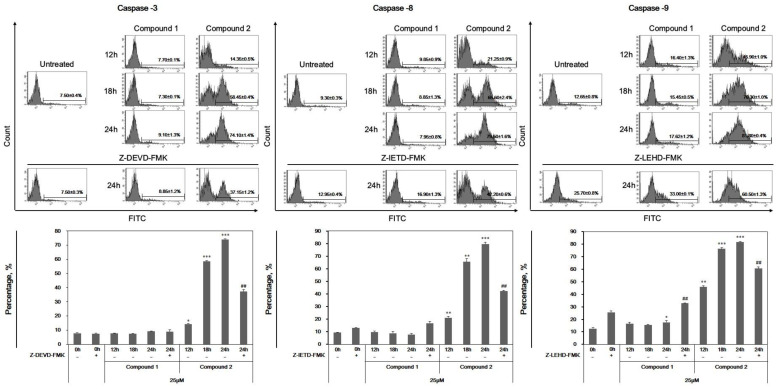
The effect of compounds **1** and **2** on the activation of caspase-3, -8, and -9. HL-60 cells were treated with the compounds for 12, 18, and 24 h. After the indicated incubation times, cells were harvested and stained with FITC for 1 h. Activated caspase-8 was analyzed by flow cytometry. The results are presented as the means ± S.D. of experiments performed in duplicate. * *p* ≤ 0.05, ** *p* ≤ 0.01, *** *p* ≤ 0.001, ## *p* ≤ 0.0001.

Caspases, a family of cysteine proteases, are known to play an integral role in the apoptotic pathway. Therefore, we investigated whether compound **2** induced apoptosis in leukemia cells via caspase activation by analyzing the expression of activated caspase-3, -8, and -9. As shown in Figure 8, activated caspases-3, -8, and -9 increased in a time-dependent manner in response to compound **2**, similar to the effects of each caspase inhibitor (Z-DEVD-FMK; caspase-3 inhibitor, Z-IETD-FMK; caspase-8 inhibitor, Z-LEHD-FMK; caspase-9 inhibitor). These suggest that compound **2** induces MMP collapse, leading to the activation of caspase.

## 4. Discussion

Despite advances in cancer treatment, it remains one of the most common causes of death. Cancer therapies typically involve the surgical resection of tumors, followed by irradiation and the use of antitumor agents. Anticancer drugs are also administered as first-line therapy to induce apoptosis in cancer cells. However, many synthetic agents also induce apoptosis in normal cells, often causing significant adverse effects. Recently, research on the development of new antitumor agents has increasingly focused on herbal medicine. Herbal medicine has been used for a long time and is known for its low side effects.

*P. japonicum* is a perennial herb distributed in Japan, the Philippines, China, and Korea, and its roots are traditionally used to treat coughs, colds, and headaches [5]. However, the anticancer effects of its roots have not been explored to date. The active compounds **1** and **2**, isolated from *P. japonicum* in the present study, possess the same core structure but differ in their side chain and stereochemistry at C-3′ and C-4′. This study shows that these compounds exhibit different cytotoxicities against the leukemia cell line HL-60. The senecioyl group, which is a practical functional group, can be identified in the structure of compound **2** and has been shown to inhibit inflammation [36]. In addition, research has confirmed that the senecioyl group present in *Angelica keiskei* roots is responsible for inducing apoptotic death in HL-60 cells [37]. Onder reported that coumarin-type compounds obtained from *Zanthoxylum schinifolium* significantly inhibited the proliferation of HL-60 human acute promyelocytic leukemia cells, with IC₅₀ values ranging from 4.62 to 5.12 μM [38]. Remarkably, coumarins possess potent antiproliferative activity, which suppresses cell growth and induces apoptosis in various cancer cells including HL-60 (leukemia), A549 (lung), ACHN (renal), H727 (lung), and MCF-7 (breast) both in vitro and in vivo [39,40]. Furthermore, coumarins have been shown to exhibit both cytotoxic and cytostatic effects [41]. In the present study, compound **2** at a concentration of 30 μM was shown to reduce the survival rates of three cancer cell lines. Interestingly, only compound **2** dramatically reduced the viability of HL-60 cells. Furthermore, we focused on the effects of both compounds on the apoptosis of HL-60 cells, mediated via cellular mechanisms involving the activation of caspase-3, -8, and -9, and observed morphological characteristics of apoptosis, such as apoptotic body formation and nuclear fragmentation. In connection to this, our results were in accordance with the findings of Wu et al., which demonstrated that coumarins can up-regulate the expression of both caspase-3 and caspase-9 proteins, as well as Bax protein, while down-regulating Bcl-2 protein expression in leukemia cell lines [42]. Compound **2** increased the proportion of cells in the sub-G1 phase while decreasing the proportions of cells in the G1/S/G2/M phases in a time-dependent manner. The number of living cells (Annexin V/PI double-negative cells) decreased in a time-dependent manner, and the percentages of cells in early and late apoptosis significantly increased upon treatment with compound **2**. The collapse of MMP is also a key indicator of apoptosis, and treatment with compound **2** promotes this collapse. Apoptosis driven by the mitochondrial pathway is characterized by plasma membrane blebbing, condensation, cell fragmentation, and the extensive degradation of chromosomal DNA [43]. Based on the observed morphological changes following treatment with compound **2**, this type of cell death is classified as apoptotic [43].

## 5. Conclusions

In the present study, two bioactive compounds, **1** and **2**, were successfully isolated from the roots of *P. japonicum*. Notably, compound **2,** at a concentration of 30 μM, demonstrated remarkable anticancer activity against the HL-60 leukemia cell line. Specifically, compound **2** (30 μM) exhibited the potent induction of cell death in HL-60 leukemia cells via the mitochondria-mediated pathway, leading to the activation of the caspase family. These findings highlight the selective apoptotic effect of compound **2** (30 μM) on leukemia cells, emphasizing its therapeutic potential. Thus, the compound isolated from the roots of *P. japonicum* presents a promising candidate for the development of anticancer agents, particularly for the treatment of leukemia.

## Data Availability

The datasets used and/or analyzed during the current study are available from the corresponding author upon reasonable request.

## Supplementary Materials

The following supporting information can be downloaded at: https://www.mdpi.com/article/10.3390/cells13231982/s1. Figure S1: The *P. japonicum* roots separation process. Figure S2: HPLC chromatogram of (-)-isosamidin (**1**) and 3′S,4′S disenecioylkhellactone (**2**). Figure S3: Mass spectra data of (-)-isosamidin (**1**). Figure S4: Mass spectra data of 3′S,4′S disenecioylkhellactone (**2**). Figure S5: ^1^H NMR spectrum of (-)-isosamidin (**1**) in CDCl_3_. Figure S6: ^1^H-^1^H COSY spectrum of (-)-isosamidin (**1**) in CDCl_3_. Figure S7: HSQC NMR spectrum of (-)-isosamidin (**1**) in CDCl_3_. Figure S8: HMBC NMR spectrum of (-)-isosamidin (**1**) in CDCl_3_. Figure S9: ^1^H NMR spectrum of 3′S,4′S disenecioylkhellactone (**2**) in CDCl_3_. Figure S10: ^1^H-^1^H COSY spectrum of 3′S,4′S disenecioylkhellactone (**2**) in CDCl_3_. Figure S11: HSQC NMR spectrum of 3′S,4′S disenecioylkhellactone (**2**) in CDCl_3_. Table S1: NMR data for **1**–**2** in CDCl_3_.
