# Supplementary material for: Evaluation of Apoptosis-Inducing Coumarins Isolated from Peucedanum japonicum Roots: The Potential for Leukemia Treatment via the Mitochondria-Mediated Pathway
Source: Cells. 2024 Nov 29;13(23):1982. doi: 10.3390/cells13231982 (PMC11639758; doi:10.3390/cells13231982)
Supplement: Supplementary file 1 [file cells-13-01982-s001.zip › cells-3311619-supplementary.pdf]

## Supplementary Materials

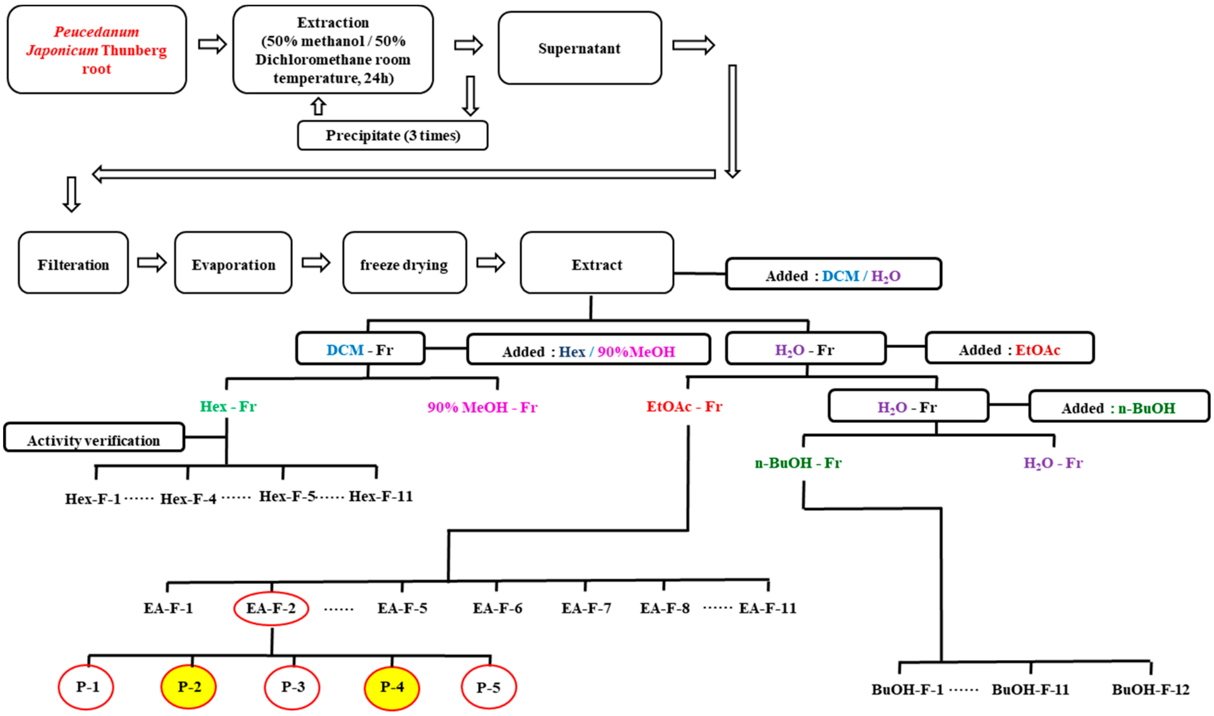


**Figure S1.** The *P. japonicum* roots separation process.

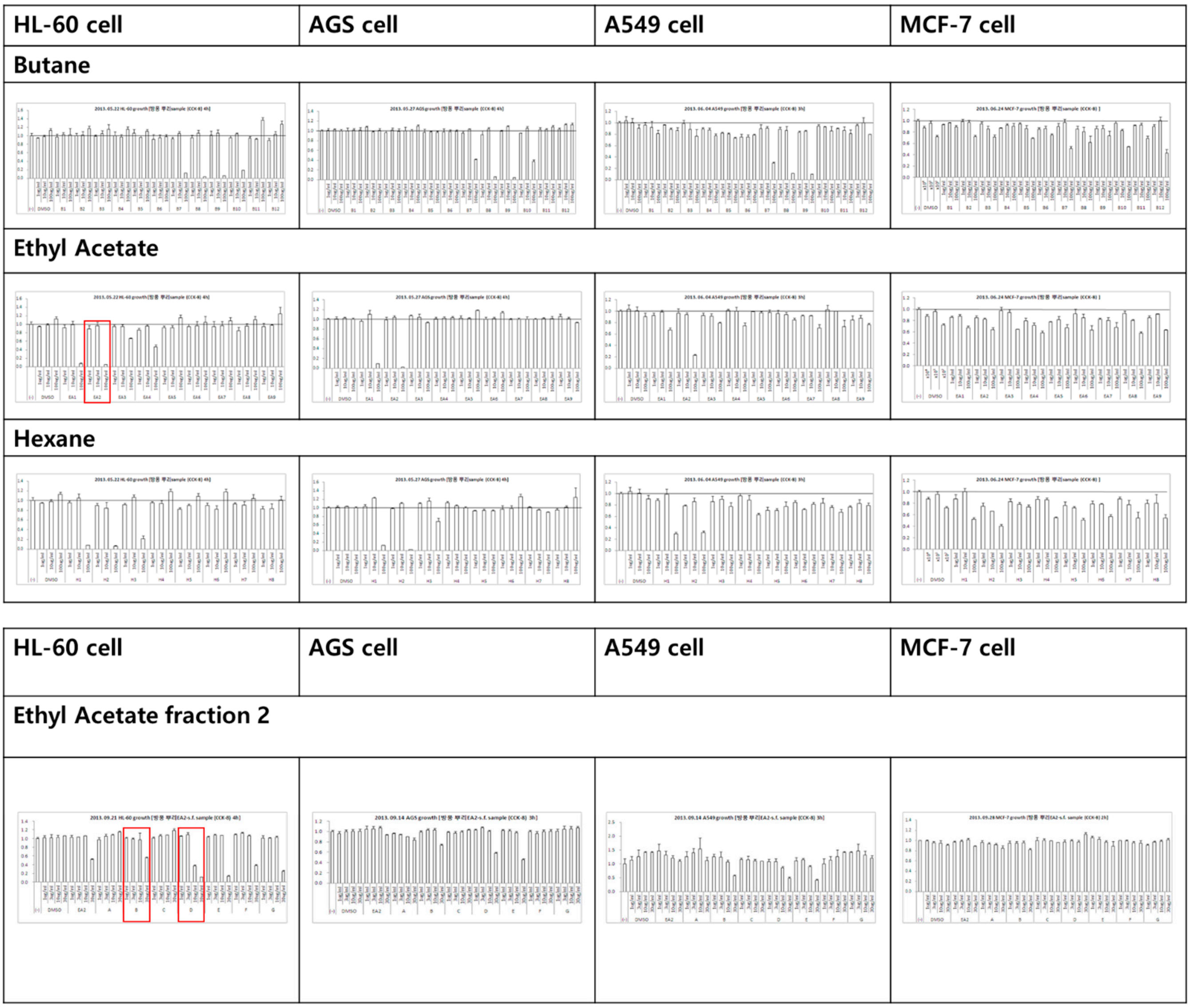


**Figure S2.** Process of isolating compounds **1** and **2**.

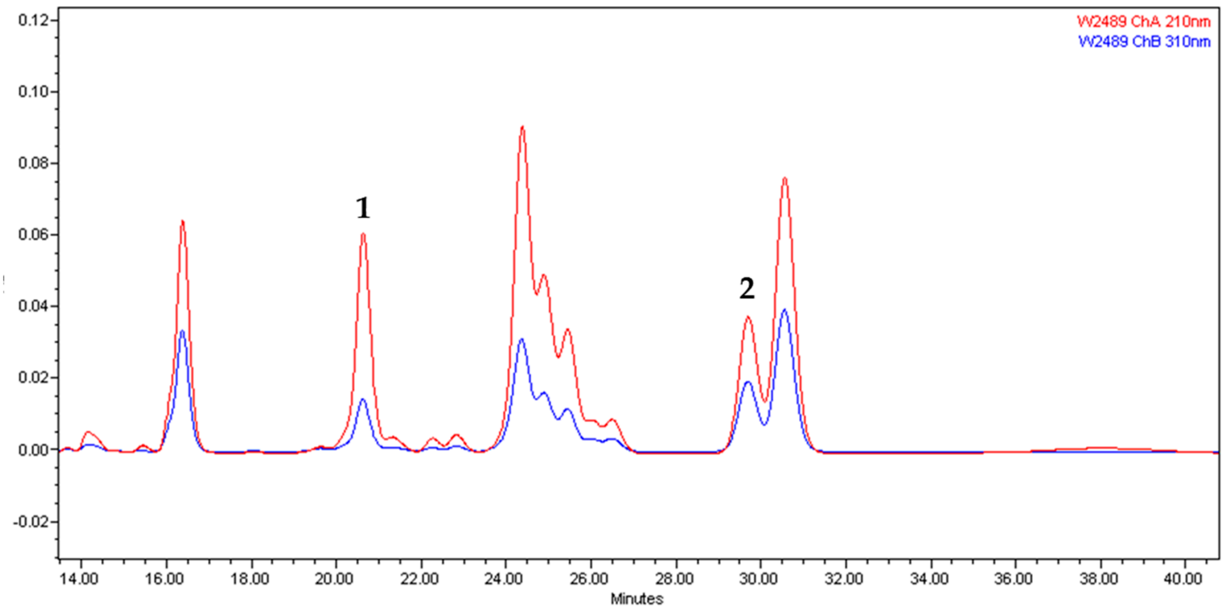


**Figure S3.** HPLC chromatogram of (-)-isosamidin (**1**) and 3'*S*,4'*S*-disenecioylkhellactone (**2**).

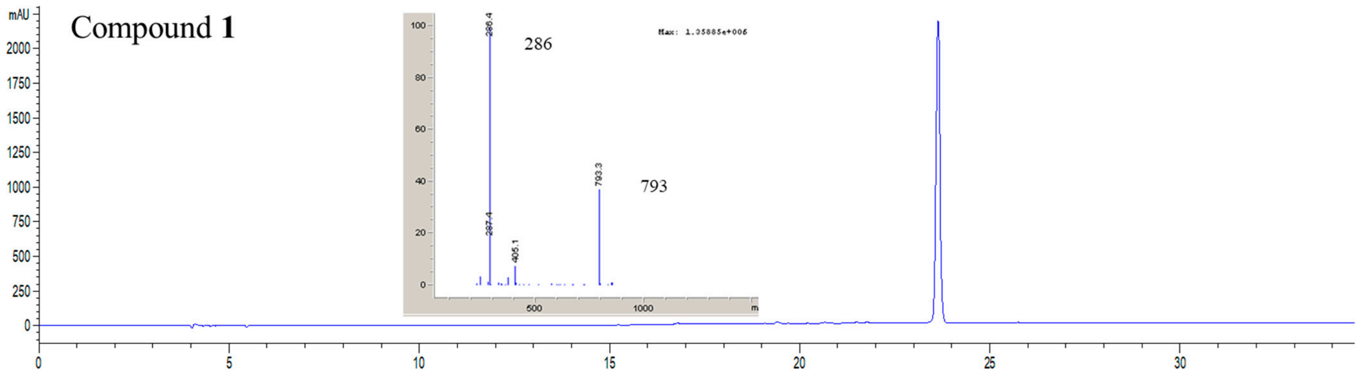


**Figure S4.** Mass spectra data of (-)-isosamidin (**1**).

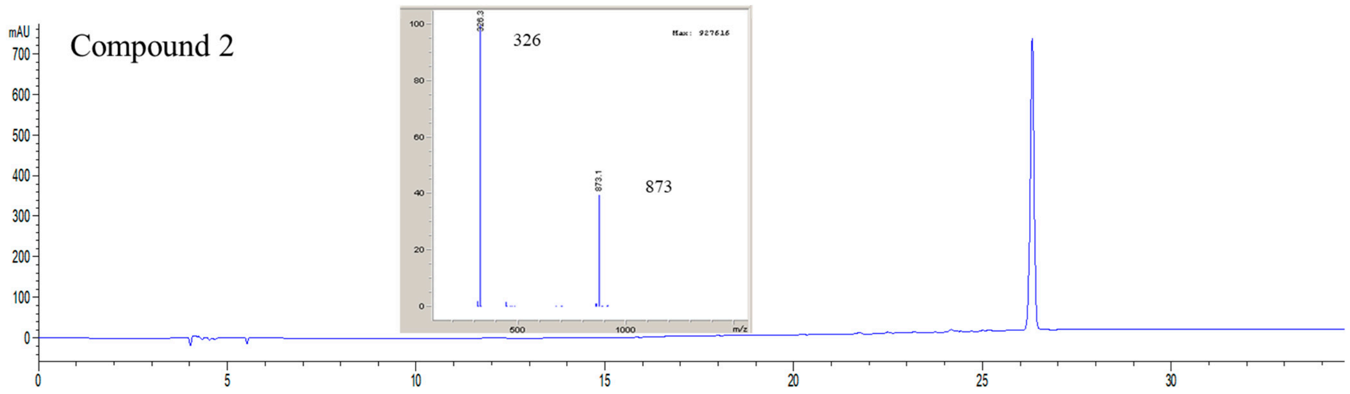


**Figure S5.** Mass spectra data of 3'*S*,4'*S*-disenecioylkhellactone (**2**).

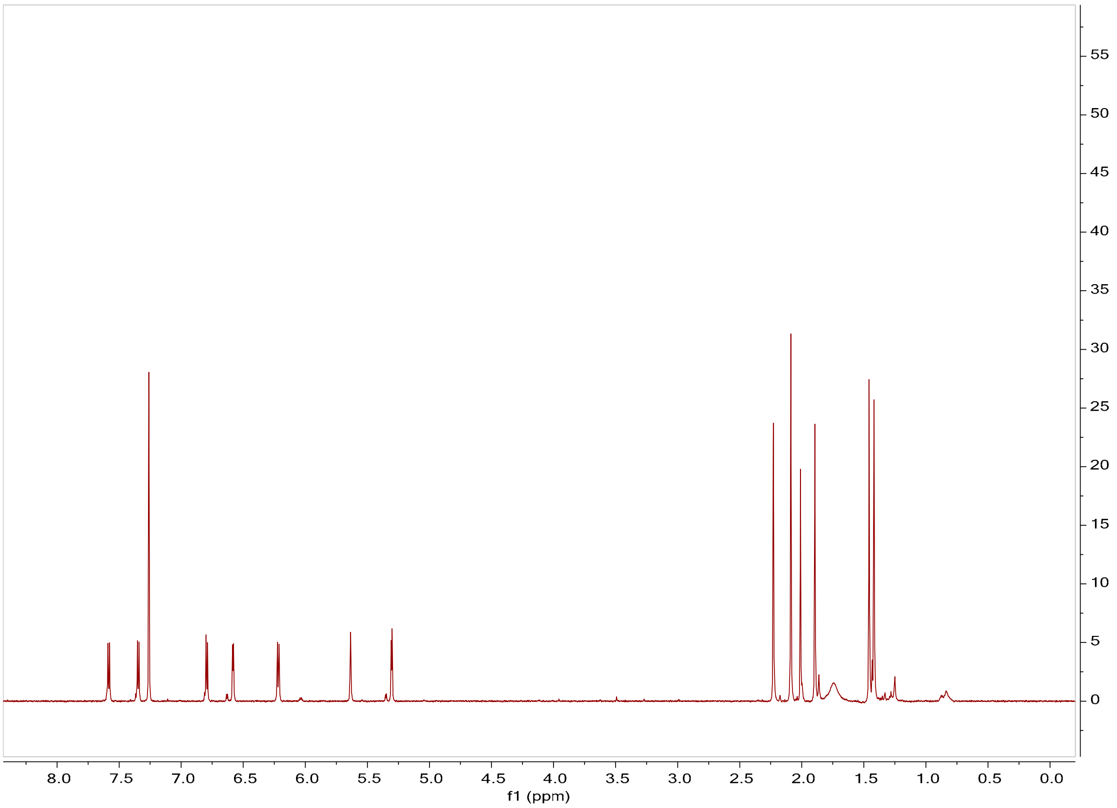


**Figure S6.** $^{1}H$ NMR spectrum of (-)-isosamidin (**1**) in $CDCl_3$

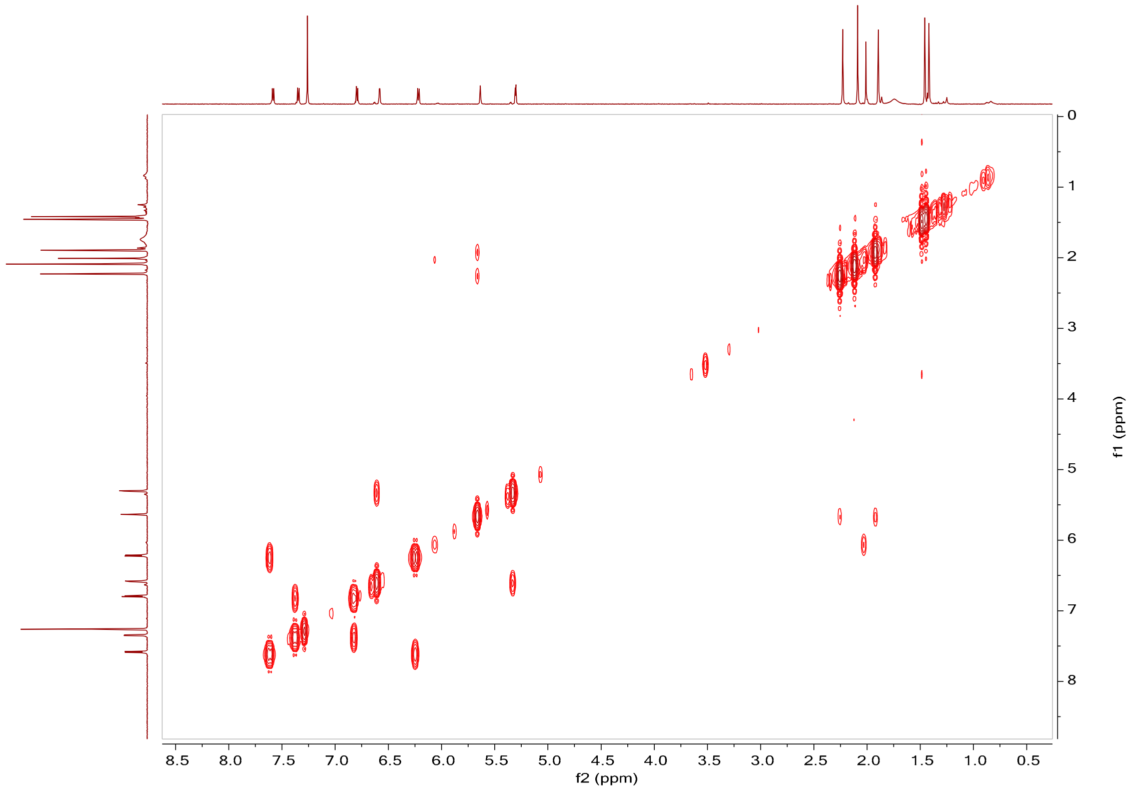


**Figure S7.** $^{1}H$-$^{1}H$ COSY spectrum of (-)-isosamidin (**1**) in $CDCl_3$.

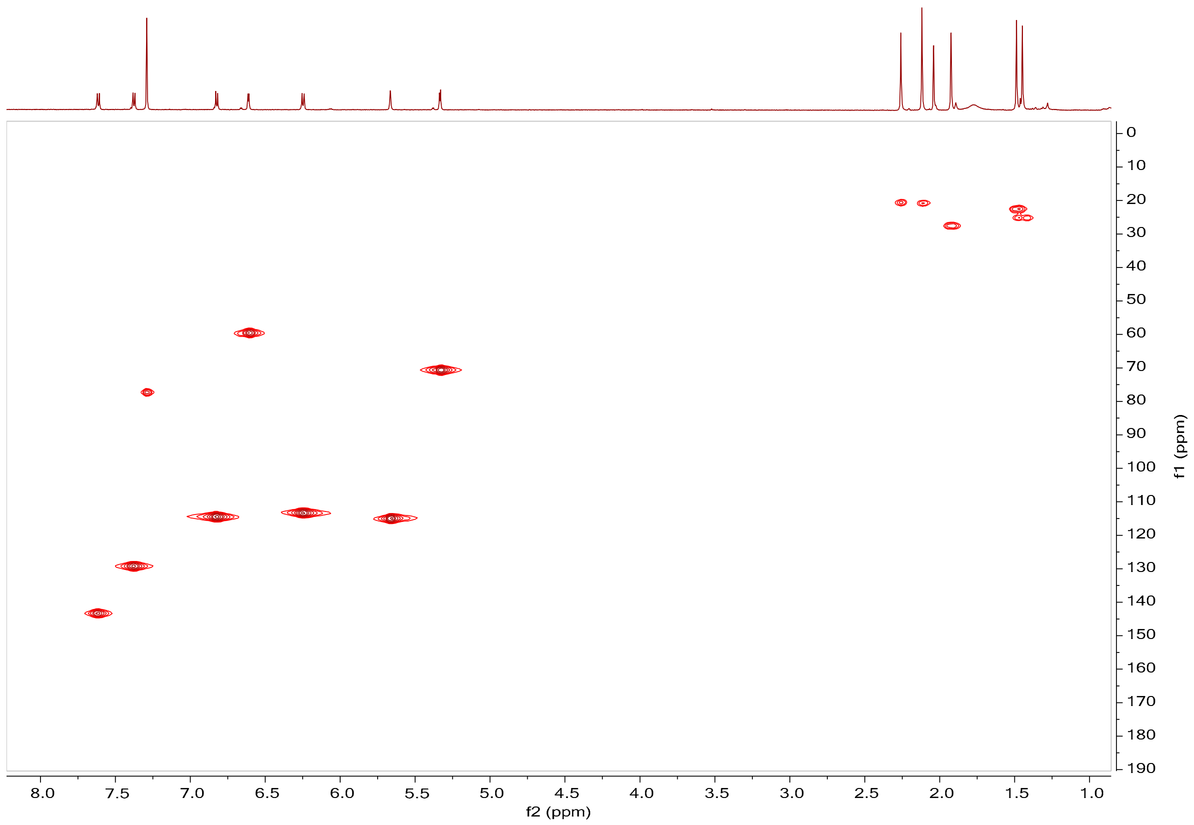


**Figure S8.** HSQC NMR spectrum of (-)-isosamidin (**1**) in $CDCl_3$.

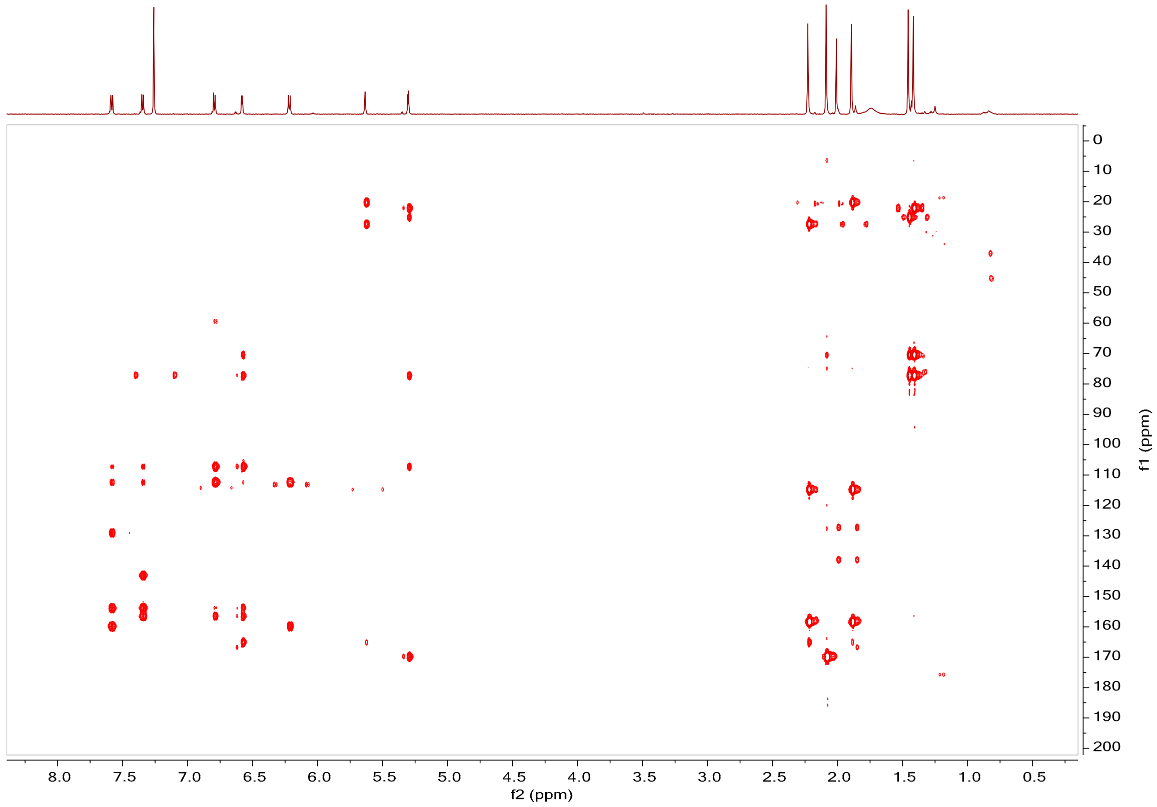


**Figure S9.** HMBC NMR spectrum of (-)-isosamidin (**1**) in $CDCl_3$..

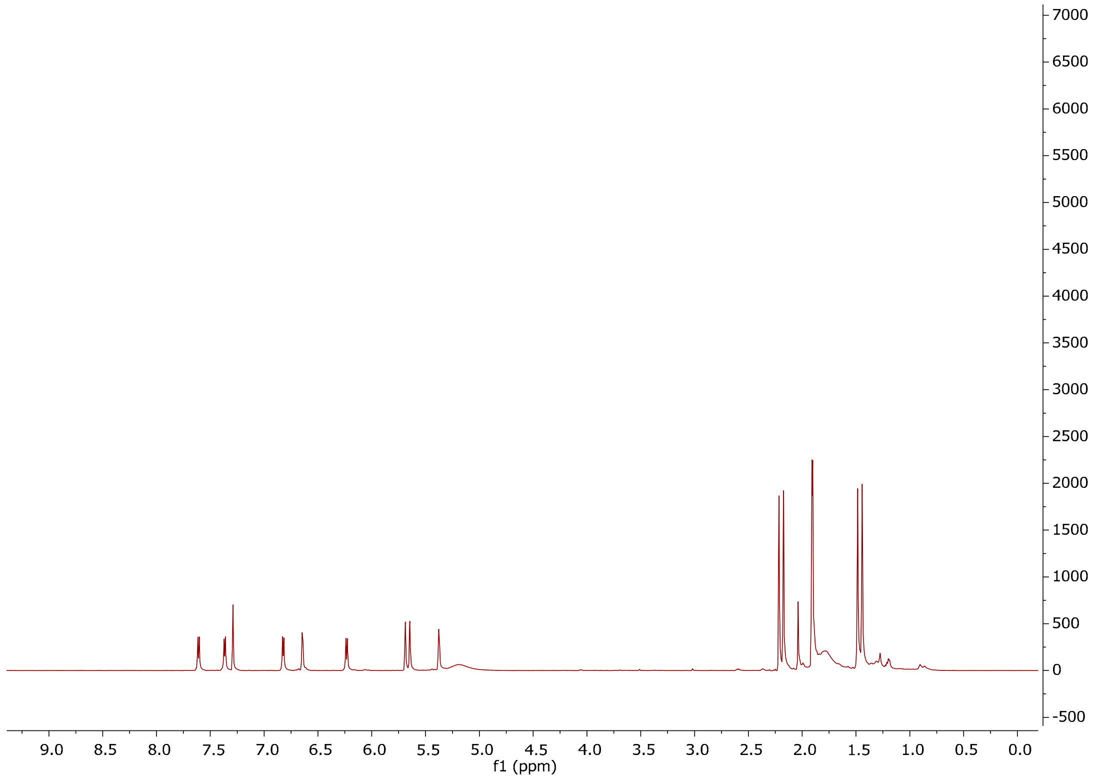


**Figure S10.** $^1$H NMR spectrum of 3'*S*,4'*S*-disenecioylkhellactone (**2**) in $CDCl_3$

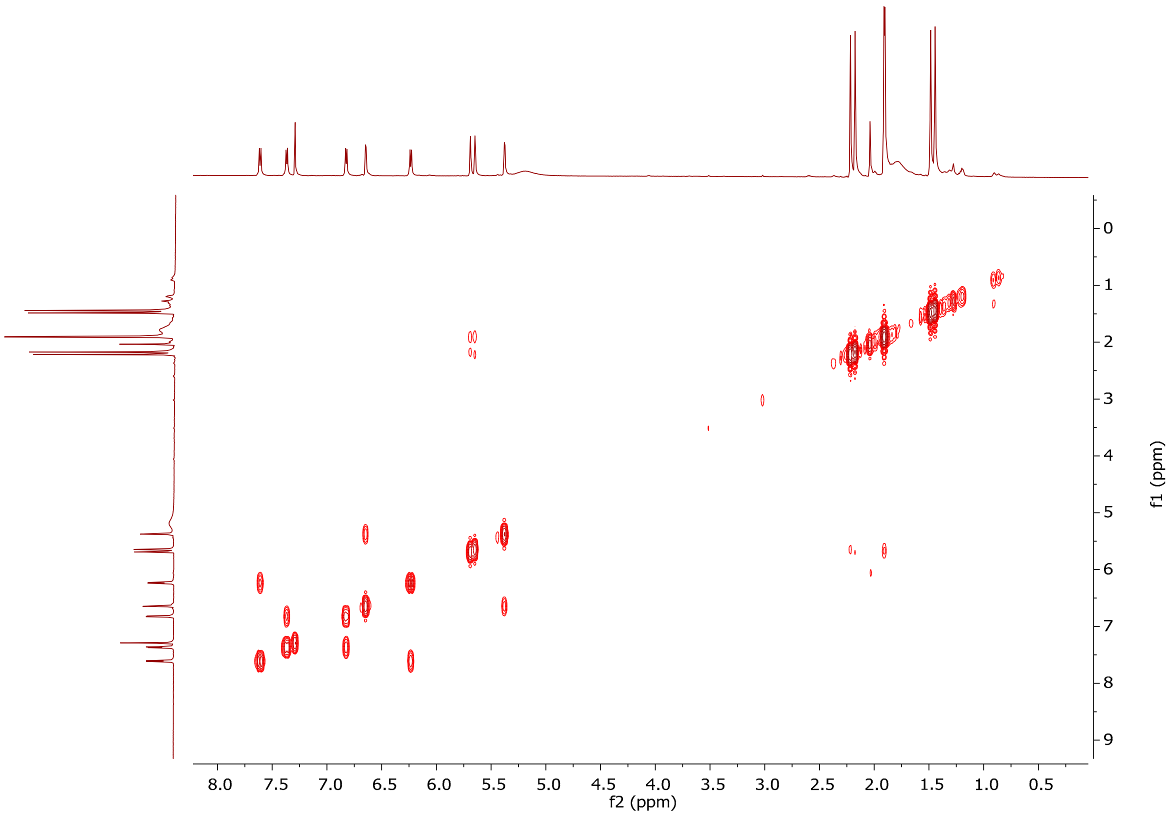


**Figure S11.** $^1$H-$^1$H COSY spectrum of 3'*S*,4'*S*-disenecioylkhellactone (**2**) in $CDCl_3$

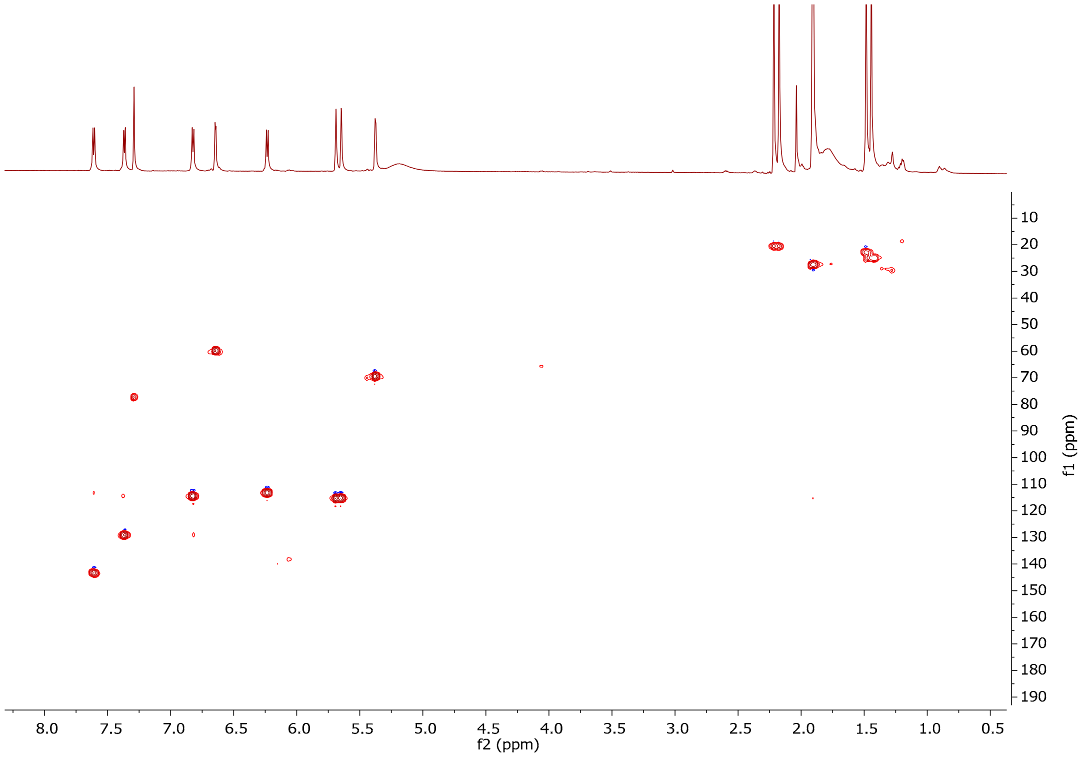


**Figure S12.** HSQC NMR spectrum of 3'*S*,4'*S*-disenecioylkhellactone (**2**) in $CDCl_3$

**Table S1.** NMR data for compounds **1** and **2** in $CDCl_3$.

| No. | **1** | | **2** | |
|---|---|---|---|---|
| | $\delta_C$*, type | $\delta_H$, (*J* in Hz) | $\delta_C$*, type | $\delta_H$, (*J* in Hz) |
| **2** | 159.5, C | | 162.7, C | |
| **3** | 112.8, CH | 6.23, d (9.5) | 114.4, CH | 6.24, d (9.5) |
| **4** | 143.2, CH | 7.61, d (9.5) | 146.6, CH | 7.60, d (9.2) |
| **5** | 128.7, CH | 7.36, d (8.7) | 131.7, CH | 7.37, d (8.7) |
| **6** | 114.1, CH | 6.81, d, (8.5) | 114.8, CH | 6.82, d, (8.7) |
| **7** | 156.4, C | | 158.8, C | |
| **8** | 106.7, C | | 109.4, C | |
| **9** | 153.6, C | | 155.9, C | |
| **10** | 112.3, C | | 113.6, C | |
| **2′** | 77.7, C | | 79.4, C | |
| **3′** | 68.7, CH | 5.35, d (4.8) | 72.0, CH | 5.37, d (4.8) |
| **4′** | 59.5, CH | 6.57, d (4.8) | 61.8, CH | 6.65, d (4.8) |
| **5′** | 24.5, $CH_3$ | 1.41, s | 26.5, $CH_3$ | 1.44, s |
| **6′** | 23.1, $CH_3$ | 1.45, s | 23.2, $CH_3$ | 1.48, s |
| **Acetyl** | 169.5, C | | | |
| | 20.6, $CH_3$ | 2.11, s | | |
| **Senecioyl** | 164.9, C | | 167.5, C | |
| | 114.8, CH | 5.69, s | (167.3, C) | |
| | 158.4, C | | 117.4, CH | 5.64, s |
| | 27.4, $CH_3$ | 1.90, s | (117.5, CH) | (5.69, s) |
| | 20.3, $CH_3$ | 2.22, s | 160.8, C | |
| | | | (160.0, C) | |
| | | | 28.3, $CH_3$ | 1.91, s |
| | | | (28.2, $CH_3$) | (1.90, s) |
| | | | 21.2, $CH_3$ | 2.22, s |
| | | | (21.3, $CH_3$) | (2.17, s) |

*Carbon signals were assigned from the 2D NMR data analysis.
